# Traditional Health Practices May Promote Nrf2 Activation Similar to Exercise

**DOI:** 10.3390/ijms262311546

**Published:** 2025-11-28

**Authors:** Hubert Kolb, Stephan Martin, Kerstin Kempf

**Affiliations:** 1 West-German Centre of Diabetes and Health, Düsseldorf Catholic Hospital Group, 40591 Düsseldorf, Germany; 2 Faculty of Medicine, Heinrich-Heine-University Düsseldorf, 40225 Düsseldorf, Germany

**Keywords:** caloric restriction, cryotherapy, cupping, exercise, hyperbaric oxygen, hypoxia, polyphenols, sauna

## Abstract

Various non-pharmacological practices have been reported to enhance overall health. The molecular effects of exercise have been shown to involve the upregulation of enzymes and transcription factors that enhance antioxidative and anti-inflammatory activity, boost mitochondrial function and growth, and promote a parasympathetic tone. These beneficial changes occur as an adaptive/hormetic response to an initial increase in oxygen radical and nitric oxide production in working muscles. The redox-sensitive nuclear factor erythroid 2-related factor 2 (Nrf2) was identified as the key mediator of the cellular defense response. A similar adaptive response appears to occur in response to exposure to heat or cold, hyperbaric or hypobaric oxygen, cupping therapy, acupuncture, caloric restriction, and the consumption of polyphenol-rich plant-based foods or spices, and there is direct or indirect evidence for the involvement of Nrf2. In many cases, additional stress signaling pathways have been observed to be upregulated, including the nicotinamide adenine dinucleotide (NAD+)-sirtuin and the adenosine monophosphate (AMP)-activated protein kinase pathways. We conclude that while several traditional health practices may share a hormetic mechanism—mild radical-induced damage triggers a defense response through upregulation of antioxidative, anti-inflammatory, and repair activities, which may impact body-wide tissue function.

## 1. Introduction

Physical exercise has been widely recognized for its ability to enhance physiological functions and extend lifespan in both animals and humans, leading to its classification as “medicine” [1,2,3,4,5,6]. Benefits of exercise are observed not only in tissues directly engaged, such as skeletal muscle and the heart, but also in distant tissues like the central nervous system and pancreatic islet beta cells [7,8,9,10]. Thus, the working muscles not only adapt to increased physical demands by improving their functional capacity but also trigger a systemic beneficial response.

Similar body-wide benefits are claimed—though less extensively studied—for various practices, many of which have been employed for centuries to strengthen the body or restore health. These practices include exposure to heat or cold, hyperbaric or hypobaric oxygen, cupping therapy, acupuncture, caloric restriction, and the consumption of polyphenol-rich plant-based foods or spices, ayurveda, yoga, mindfulness-based stress reduction, meditation, breathwork, Tai Chi, Qigong, and massage therapy.

The general health claims associated with these non-pharmacological treatments largely overlap with those documented for regular physical exercise. In this paper, we examine the limited available data on the molecular health mechanisms of traditional lifestyle practices. We propose that many health-promoting practices may share an underlying molecular mechanism with physical exercise.

## 2. Materials and Methods

A literature search was conducted in PubMed and Medline databases with the aim of identifying traditional non-pharmacological health practices where some information was available regarding the molecular effects of the health intervention. Key terms for molecular effects included “oxygen radicals/oxidative stress”, “inflammation”, “Nrf2”, “sirtuin”, “AMP-activated protein kinase”. Traditional non-pharmacological health practices included exercise, exposure to heat or cold, hyperbaric or hypobaric oxygen, cupping therapy, acupuncture, caloric restriction, the consumption of polyphenol-rich plant-based foods or spices, ayurveda, yoga, mindfulness-based stress reduction, meditation, breathwork, Tai Chi, Qigong, and massage therapy. Both human and animal studies were included. Where available, meta-analyses were selected in preference to individual trial data. Information on molecular effects was sometimes only available from animal models. Only articles published in English were selected.

## 3. Physical Exercise

**Figure 1:**
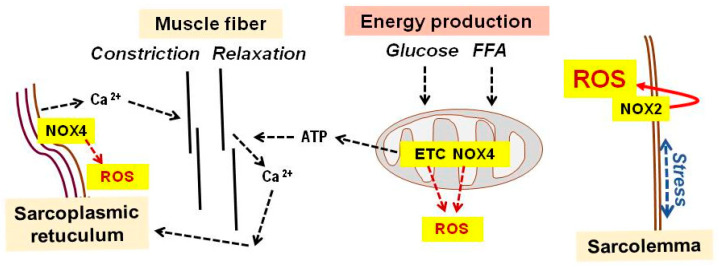
Oxygen radical production by the working muscle. The contraction and relaxation of muscle fibers during exercise is driven by calcium ions released from the sarcoplasmic reticulum and their adenosine triphosphate (ATP)-dependent return. The dominant source of oxygen radicals is sarcolemma-bound nicotinamide adenine dinucleotide phosphate oxidases (NOX)-2, activated by the mechanical stress of the plasma membrane during muscle work. Additional sources of reactive oxygen species (ROS) are NOX4 in the sarcoplasmic reticulum and the electron transfer chain, as well as NOX4 in mitochondria, among others. → defines “comes from” or “acts on”. ETC, electron transfer chain; FFAs, free fatty acids.

A bout of exercise initially induces cellular and systemic damage while simultaneously activating the sympathetic nervous system. During calcium-dependent muscle contraction and adenosine triphosphate (ATP)-mediated relaxation, superoxide production increases, primarily from nicotinamide adenine dinucleotide phosphate (NADPH) oxidases (NOX) outside mitochondria. Repeated exercise leads to additional superoxide production via the electron transfer chain and NOX within mitochondria (Figure 1) [11,12,13,14,15,16].

Membrane-bound NOX2 is the major source of superoxide during skeletal muscle contraction, activated by membrane depolarization and mechanical stretching of the sarcolemma [17,18,19]. Superoxide is converted into hydrogen peroxide and other reactive oxygen species (ROS), which damage proteins, lipids, and other components of muscle tissue and blood [13,20]. Nitric oxide (NO) production also increases during exercise, primarily through neuronal NO synthase (NOS) [14]. Thus, muscle activity comes at the cost of elevated ROS and NO levels and the resulting oxidative and nitrosative damage (Figure 1).

There is no clear physiological reason for increased radical production from NADPH oxidases in working muscles, as this process is not directly linked to ATP production within mitochondria. Instead, high-intensity exercise appears to reduce oxygen radical leakage from the electron transfer chain (ETC) in mitochondria, but there may be mitochondrial leakage in muscles of less well-trained yet recreationally active persons [15,21]. The increased radical challenge during exercise, coupled with sympathetic nervous system activation, serves as a signal triggering an adaptive response in muscle tissue. This response is characterized by improved radical defense mechanisms and enhanced repair activity, along with parasympathetic nervous system upregulation to counteract sympathetic activity. These molecular adaptations are the foundation of the health benefits observed following repeated exercise [14,22]. The expression of major antioxidant enzymes is upregulated by 20–180%, and exercise-induced oxidative damage to cellular proteins and lipids is significantly reduced [14,20,23,24]. Another key component of the adaptive response is the activation of mitochondrial growth, leading to physiological changes that improve muscle performance—commonly referred to as the training effect [25,26]. Upregulated antioxidant enzymes include superoxide dismutases, glutathione peroxidases, peroxiredoxins, thioredoxins, and catalase [13]. This antioxidant response also provides protection against other types of stress, including mechanical (eccentric) and toxic stress [14].

During exercise, extracellular superoxide dismutase is also upregulated in muscle tissue. This antioxidant enzyme binds to cell surfaces in distant regions via its heparin-binding domain, acting as a body-wide protective agent that helps prevent oxidative stress-related diseases, such as lung or ischemic heart disease [27]. Since exercise-induced ROS levels also rise in the bloodstream, a systemic adaptive defense response is triggered, evidenced by the upregulation of antioxidant enzymes and enhanced resistance to radical stress, as observed in tissues such as the testes and liver [28,29].

There is compelling evidence that the adaptive antioxidative response is triggered by the action of ROS, primarily hydrogen peroxide (H_2_O_2_), which is derived from the superoxide anion radical. Studies involving animals and humans have shown that treatment with antioxidant vitamins C and E reduces ROS levels and mitigates oxidative damage [30]. However, this intervention also blunts the training effects typically observed with regular exercise. Suppressing superoxide formation exclusively from NADPH oxidases 2 and 4 similarly inhibits the training effect, with NOX2 being more influential in skeletal muscle and NOX4 playing a key role in the heart. Additionally, blocking NO formation with a NOS inhibitor attenuates the training effect [30]. These findings indicate that the damaging effects of increased ROS and NO levels are essential for initiating the beneficial adaptive response in muscle tissue and other organs following exercise [15,31,32,33,34].

The electrophilic attack of ROS and NO primarily targets the sulfhydryl groups of cysteine, and there is lipid peroxidation and deoxyribonucleic acid (DNA) oxidation. While many proteins are functionally affected by the oxidation or nitrosation of cysteine sulfhydryl (SH) groups, only a few can be considered genuine sensors for ROS or NO due to their pleiotropic effects on cellular function. These include oxidation-sensitive transcription factors, kinases, and phosphatases.

**Figure 2:**
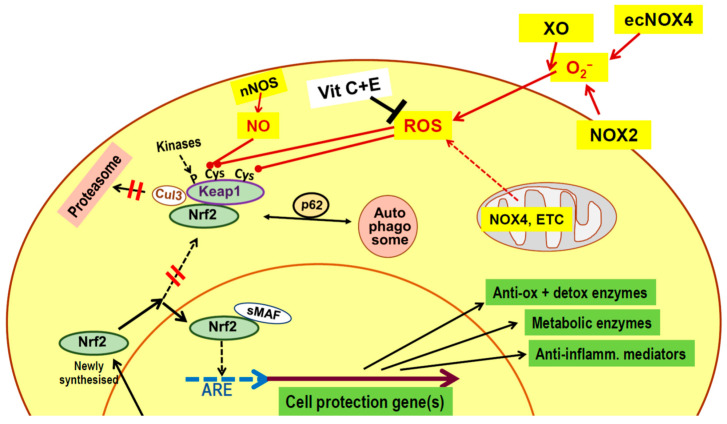
Activation of nuclear factor erythroid 2-related factor 2 (Nrf2) as an adaptive response to oxidative stress. Sulfhydryl (SH) groups of Kelch-like ECH-associated protein 1 (Keap1) are sensors of increased oxygen radicals and nitric oxide levels in the working muscle. Oxidative modification of SH-groups inhibits the ability of Keap1 to deliver continuously synthesized Nrf2 to proteasomal destruction. Therefore, newly formed Nrf2 can translocate to the nucleus, associate with small musculoaponeurotic fibrosarcoma protein (sMAF), and bind to the antioxidant response element (ARE) sequence in the promoter region of a large number of cell protective genes, which causes enhanced transcription. Ingestion of vitamin (Vit) C plus vitamin E for neutralization of radical oxygen species (ROS) preserves Keap1 functions and prevents increased expression of antioxidant enzymes, mitochondrial growth, and the training effect of exercise. → defines “comes from” or “acts on”; ⊣ defines “inhibits”. Anti-inflamm., anti-inflammatory; Anti-ox, antioxidative; Cul3, Cullin 3-based E3 ubiquitin ligase; ecNOX, endothelial constitutive NOX; ETC, electron transfer chain; detox, detoxifying; nNOS, neuronal NO synthase; NOX, NADPH oxidase; p62, protein p62; XO, xanthine oxidase.

The nuclear factor erythroid 2-related factor 2 (Nrf2)/Kelch-like ECH-associated protein-1 (Keap1) complex appears to be the primary sensor of ROS and NO in active muscle, with Nrf2 acting as the key mediator of the adaptive antioxidant response. Nrf2 promotes gene expression by binding to the antioxidant response element (ARE) present in the promoter regions of several hundred genes that code for cytoprotective enzymes, including superoxide dismutase, glutathione peroxidase, and phase II detoxifying enzymes. Additionally, Nrf2 enhances transcription of genes involved in DNA repair, protein catabolism, lipid and glucose metabolism, iron homeostasis, and anti-inflammatory activity [35,36,37,38]. Under normal conditions, Nrf2 is continuously synthesized but remains blocked by binding to Keap1, directing it toward proteasomal or autophagosomal degradation. However, elevated cytoplasmic ROS and NO levels cause oxidation/nitrosation of some SH-groups of Keap1, thereby inhibiting its ability to bind Nrf2 and preventing its degradation. Newly synthesized Nrf2 can then translocate to the nucleus, enhancing the expression of cell-protective enzymes. Additionally, phosphorylation of Keap1 by redox-sensitive kinases also interferes with channeling to the proteasome, which prevents Nrf2 turnover (Figure 2) [30,39]. The importance of Nrf2 in mediating the health effects of exercise is underscored by findings showing that blocking Nrf2 is sufficient to suppress the beneficial consequences of physical activity [30].

Mitochondrial growth in response to exercise is partially regulated by activation of the transcriptional coactivator peroxisome proliferator-activated receptor gamma coactivator-1 alpha (PGC-1α) via ROS or NO signaling. The pathway involves ROS-mediated activation of calcium/calmodulin-dependent protein kinases, AMP-activated protein kinase (AMPK), and protein p38. PGC-1α subsequently activates nuclear respiratory factors 1 and 2, leading to increased expression of mitochondrial transcription factor A [15,40,41]. Nrf2 also plays a role in upregulating nuclear respiratory factor 1, mitochondrial transcription factor A, and mitochondrial biogenesis, with Nrf2-deficient mice displaying impaired mitochondrial growth [30]. Another redox-sensitive transcription factor involved in this process is heat shock factor 1 (HSF1), which enhances the expression of heat shock proteins that stabilize protein conformations in active muscle cells [15,42]. Increased oxygen consumption during high-intensity exercise induces hypoxic conditions, activating hypoxia-induced factors that regulate gene expression in response to reduced oxygen availability [43,44].

During exercise, increased ATP consumption for muscle fiber relaxation and heightened protein synthesis lead to a rise in AMP and adenosine diphosphate (ADP) concentrations relative to ATP, as mitochondrial ATP supply remains limited. AMP serves as a sensor of cellular energy status, activating AMPK, which reduces anabolic processes while promoting catabolic activity to restore ATP levels [45]. The depletion of ATP in active muscle also results in the accumulation of oxidized nicotinamide adenine dinucleotide (NAD+), an essential sensor of impaired NADH regeneration—an ATP-dependent process [46,47]. This leads to increased activation of NAD+-dependent enzymes such as silencing information regulator 2-related enzymes (sirtuins), which deacetylate histones, leading to chromatin silencing and transcriptional repression. Several transcription factors and other protein targets are similarly modified in their activity. Sirtuins also have additional enzymatic functions, including ADP-ribosyl transferase activity, which contributes to improved mitochondrial function and increased autophagy [48,49].

For the systemic health benefits of exercise, superoxide production in muscle also occurs in the local vasculature, leading to a widespread increase in ROS levels and subsequently triggering elevated Nrf2 expression across multiple tissues [39]. Equally important is the induction of both pro- and anti-inflammatory responses in active muscles, which can impact distant parts of the body through secreted cytokines and circulating immune cells. The primary regulator of inflammatory immune activity is the redox-sensitive nuclear factor kappa light chain-enhancer of activated B cells (NFκB). Elevated ROS levels facilitate the release of NFκB from its inhibitor, IκB, enabling the formation of the p50–65 NFκB dimer and its translocation to the nucleus. This process activates genes responsible for both pro-inflammatory and anti-inflammatory responses, as well as key antioxidant enzymes [15]. The initial pro-inflammatory response is transient, followed by a sustained anti-inflammatory phase. This transition involves the transcription of Nrf2-responsive genes that suppress NFκB, inflammasome activity, and pro-inflammatory cytokine production [50]. Increased sirtuin activity during exercise further downregulates NFκB and inflammasome activation [51].

At the level of soluble immune mediators, transient expression of interleukin (IL)-6 promotes the long-lasting secretion of anti-inflammatory myokines, including IL-10, IL-1 receptor antagonist (IL-1RA), and irisin [52,53,54,55]. These anti-inflammatory mediators contribute to the systemic health benefits of exercise by counteracting the metabolic consequences of low-grade inflammation in adipose tissue, such as insulin resistance and mitochondrial dysfunction. Similar improvements are observed in various age-related inflammatory conditions, including dementia, depression, and arthritis [6,50,55,56].

Animal studies have demonstrated that various forms of exercise—such as aerobic and resistance training, but not low-intensity activity—induce upregulation of Nrf2 and activate the antioxidant defense response [57,58]. Accordingly, in this overview, the term ‘exercise’ is used in a broad sense to encompass these effective modalities.

**Figure 3:**
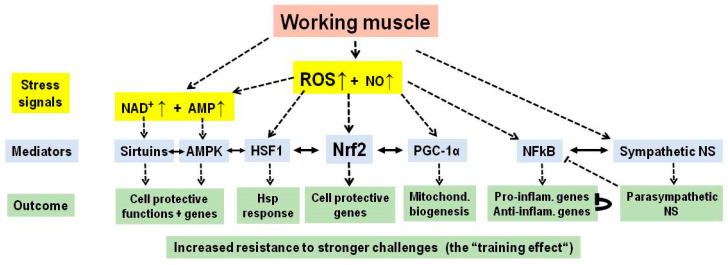
Overview of the adaptive response in the working muscle. Mechanical and metabolic stress leads to an increase in stress signals (including reactive oxygen species (ROS), nitric oxide (NO), nicotinamide adenine dinucleotide (NAD^+^), adenosine monophosphate (AMP), increased sympathetic tone) which induces a cell protective response by engaging silencing information regulator 2-related enzymes (sirtuins), AMP-activated protein kinase (AMPK), heat shock factor 1 (HSF1), nuclear factor erythroid 2-related factor 2 (Nrf2), peroxisome proliferator activated receptor gamma coactivator-1 alpha (PGC1α), nuclear factor kappa light chain-enhancer of activated B cells (NFκB) and the parasympathetic nervous system. The transcription factor NFκB elicits a transient pro-inflammatory response followed by a longer-lasting anti-inflammatory state. → defines “comes from” or “acts on”. Hsp, heat shock protein; inflam., Inflammatory; Mitochond., mitochondrial; NS, nervous system.

Taken together, a bout of exercise induces molecular damage to muscle tissue via oxygen and nitric oxide, in the context of mechanical stress and increased ATP consumption. Key characteristics include elevated levels of ROS and NO, a decreased ATP/AMP ratio, a reduced NADH/NAD+ ratio, and heightened pro-inflammatory activity. The primary mechanism behind the health benefits of exercise is the adaptive response of muscle tissue to these stressors—specifically, the induced expression or activation of enzymes responsible for radical defense, damage repair, inflammation suppression, and further enhancements such as mitochondrial growth and myogenesis. These adaptations allow muscle tissue to endure greater physical challenges than before, leading to the training effect (Figure 3). Notably, similar adaptive responses occur in organs distant from muscle tissue, likely due to the systemic circulation of damaging factors like ROS or pro-inflammatory mediators, or through the widespread effects of anti-inflammatory compounds such as extracellular superoxide dismutase and anti-inflammatory mediators activated in response to initial stress.

The adaptive defense response of cells to damage is commonly referred to as a hormetic response. Hormesis describes the phenomenon in which a moderate toxic challenge induces an adaptive response, enabling cells or organisms to tolerate significantly higher doses of the same toxin. Minimal toxin exposure may not trigger cellular damage or a subsequent protective response, while excessively high doses can overwhelm cellular defense mechanisms, leading to severe physiological impairment or even lethality [59]. Exercise exemplifies this hormetic principle: the training effect requires more than minimal exertion, whereas excessive exercise can result in long-lasting physiological dysfunction. For example, a week of high-intensity muscle exertion has been found to cause mitochondrial impairment, reduced Nrf2 protein levels, and impaired glucose tolerance. Partial recovery occurs in the subsequent week, suggesting a delayed adaptive response [60]. Thus, even extreme exercise does not entirely suppress hormesis [61].

## 4. Heat or Cold Treatment

Exposure to heat, such as sauna sessions, or cold temperatures, such as immersion in cold water, has been observed to enhance health—provided that the treatment is not excessive (as discussed below). Sauna visits or hot water bathing have been reported to improve cardiac and endothelial function for several hours following heat exposure, as well as lower blood pressure after multiple sessions [62,63]. Large prospective observational studies indicate a dose-dependent correlation between regular sauna use and a reduced risk of cardiovascular disease, along with increased health span [64]. After 20 min in a Finnish sauna, heat stress occurs, as evidenced by an elevated core body temperature of approximately 38 °C (100.4 °F). This prompts activation of the sympathetic nervous system, exerting stress on the cardiovascular system, including anterograde shear stress and an increase in heart rate by roughly 30 beats per minute [62,65,66,67,68]. Markers of oxidative stress have been observed in muscle and vascular tissue. Simultaneously, several key stress-response pathways are activated: the ROS-HSF1 axis, NAD+-sirtuin axis, AMP-AMPK axis, ROS/NO-Nrf2 axis, ROS/NO-PGC1α axis, and the ROS/NO-NFκB axis with an initial pro-inflammatory reaction, followed by a sustained anti-inflammatory response characterized by increased systemic levels of IL-1RA and IL-10 (Table 1) [62,68,69,70,71]. The initial activation of the sympathetic nervous system (SNS) is subsequently regulated by the parasympathetic nervous system (PNS) [72]. This indicates that sauna sessions induce an adaptive response similar to that observed with exercise. Although less extensively studied, other forms of sauna bathing or hot water immersion appear to elicit comparable stress responses and adaptations [62,63,64].

Cold treatment for general health and well-being is typically conducted through full-body immersion in cold water at temperatures below 15 °C (59 °F) for at least five minutes [73,74]. Other forms of cryotherapy, such as localized ice application or exposure to cold air for pain relief or post-exercise recovery, are beyond the scope of this discussion. Whole-body cold immersion initiates a cold shock response within the first few minutes, marked by activation of the sympathetic nervous system. This reaction includes transient hyperventilation, cutaneous vasoconstriction, increased heart rate, elevated blood pressure, and heightened levels of cortisol and catecholamines in the bloodstream. Thermogenesis is activated, primarily mediated by brown adipose tissue and muscle shivering, leading to an increase in basal metabolic rate by more than threefold [73,74]. Cold water immersion and winter swimming induce acute oxidative stress, as demonstrated by increased concentrations of oxidized glutathione and decreased levels of uric acid and ascorbic acid in the blood. However, increases in pro-inflammatory cytokine concentrations are either transient or absent [75,76,77]. Repeated exposure to cold water elicits a pronounced adaptive response, characterized by parasympathetic nervous system reactivation, enhanced sirtuin and AMP-activated protein kinase activity, elevated Nrf2 levels, increased antioxidant enzyme expression, and heightened anti-inflammatory activity [75,78]. Some of these adaptive effects have only been studied in response to cold air exposure, winter swimmers, or animal models [79,80,81,82,83,84]. Whether thermogenesis activation in muscle and fat tissue promotes mitochondrial biogenesis remains uncertain [85,86]. Studies investigating Nrf2 activation in response to cold treatment have been conducted in animal models, including pigs and rats [81,83]. Given that cold exposure is generally associated with oxidative stress, it is reasonable to hypothesize that similar activation of Nrf2 may occur in humans. However, this has not yet been examined.

The health benefits of repeated cold-water exposure include attenuation of the sympathetic stress response and reduced diastolic and systolic blood pressure. However, controlled studies examining well-being, mood, and susceptibility to infections remain limited, preventing firm conclusions—except for observed improvements in sleep quality [74,77,87]. Although the molecular mechanisms underlying cold exposure’s health effects are less extensively researched compared to those of heat exposure, they appear to share characteristics of an adaptive (hormetic) response similar to that seen after exercise. These include activation of sirtuins, AMPK and Nrf2, enhanced antioxidant defense, mitochondrial biogenesis, anti-inflammatory activity, and parasympathetic nervous system reactivation following cold-induced sympathetic stress (Table 1).

## 5. Hyper- or Hypobaric Oxygen Treatment

Hyperbaric oxygen exposure typically takes place in breathing chambers containing 100% oxygen at a pressure of 2–3 atmospheres. This process increases the concentration of dissolved oxygen in the body by approximately 20-fold. One significant physiological impact is enhanced wound healing and tissue regeneration. Additionally, hyperbaric oxygen treatment is utilized to support general well-being and is often promoted as a potential anti-aging therapy [88,89,90].

The immediate consequence of tissue hyperoxia is increased ROS formation from mitochondria, NADPH oxidases, and other sources [91,92]. This process is similar to the ROS production observed during exercise. The body’s adaptive response to hyperoxia also mirrors that seen after physical activity. The primary pathway involves the activation of Nrf2, which enhances the transcription of genes responsible for antioxidative defense and cell repair and regeneration. This process is further supported by sirtuins and AMPK, mitochondrial biogenesis, increased anti-inflammatory activity, and a shift toward parasympathetic nervous system dominance [73,88,91,93,94,95]. It is possible that the reduction in oxygen pressure following the termination of hyperbaric oxygen exposure contributes to the induction of this adaptive response, as cells may perceive it as decreased oxygen availability, leading to activation of hypoxia-inducible factor 1 alpha (HIF-1α) and Nrf2 [96].

Hypoxia exposure occurs in high-altitude (hypobaric) environments or in artificially controlled low-oxygen conditions (normobaric hypoxia). Restricting oxygen supply poses risks to brain function and other vital organs. In response to ambient oxygen deficiency, the body activates the sympathetic nervous system and releases stress signals. However, when hypoxia is applied at a safe dosage and duration, it can serve as a hormetic stimulus, leading to improved vascular, brain, and overall organ function, as well as increased resilience to subsequent hypoxic episodes. In most studies, intermittent hypoxia—short-term exposure to reduced oxygen levels lasting from minutes to one hour, administered 1–3 times per week—has been used for several weeks. Documented health benefits include reduced heart rate and blood pressure, along with improved cognitive function. Patients with cardiovascular, metabolic, or neurodegenerative diseases may also benefit from intermittent hypoxia treatment. Whether training in hypoxic conditions enhances athletic performance remains under debate, although studies indicate a positive effect on vascular function (see refs. [97,98,99,100]).

Since oxygen deprivation can be life-threatening, even moderate intermittent hypoxia induces cellular stress markers. In addition to sympathetic nervous system activation, NO production increases to promote vasodilation. HIF-1α activation occurs due to reduced oxygen-induced turnover, while elevated ROS formation from mitochondrial electron transport chains and NADPH oxidase 4 triggers Nrf2 activation. Simultaneously, NFκB, PGC-1α, sirtuins, and AMPK are activated [47,101,102]. Both HIF-1α and Nrf2 transcription factors translocate to the nucleus, where they initiate the expression of genes involved in the adaptive response to hypoxia and ROS-mediated cellular damage. NFκB regulates both pro- and anti-inflammatory gene transcription. Additionally, PGC-1α, sirtuins, and AMPK improve mitochondrial function alongside other cytoprotective activities (Table 1). Overall, the hormetic response to moderate hypoxia closely resembles that observed during exercise.

## 6. Fasting or Caloric Restriction

The health benefits of fasting, intermittent fasting, and caloric restriction have been extensively researched for decades, with confirmed positive effects on cardiovascular, metabolic, cognitive, and immune function [103,104]. Studies examining the molecular mechanisms behind these benefits highlight striking similarities to the signaling pathways activated by exercise, reinforcing the concept of a hormetic response in caloric restriction [105].

An acute stress response to fasting involves sympathetic nervous system activation, which gradually resolves as metabolic adaptation occurs. In cases of mild caloric restriction, cortisol levels may not significantly increase, especially in obese individuals [106,107]. Over the long term, fasting leads to parasympathetic nervous system activation and reductions in both diastolic and systolic blood pressure [108]. Similar to exercise, fasting engages key stress-response signals, including increased ROS levels, subsequent Nrf2 activation, and elevated NAD+ and AMP concentrations. These changes enhance sirtuin and AMPK activity and activate PGC-1α while simultaneously downregulating NFκB. Collectively, these mechanisms result in increased antioxidative, anti-inflammatory, mitochondrial, and cell-regenerative activity (Table 1; see refs. [104,105,109,110,111,112,113,114,115]). Although the role of a reshaped gut microbiota in these responses remains unclear, gut bacteria have demonstrated the potential to promote Nrf2 activation [116]. Regenerative processes may be induced following the reintroduction of normal caloric intake and concomitant reactivation of anabolic metabolism [117]. Notably, intermittent fasting and limited fasting periods have been practiced as part of religious and spiritual traditions for centuries [118,119].

## 7. Dietary Polyphenols and Other Phenolics

Consuming polyphenol-rich foods has been linked to reduced rates of cardiovascular, metabolic, neurodegenerative diseases, and cancer [120,121,122]. Due to the extended time required to observe clinical endpoints, randomized controlled trials on long-term polyphenol consumption are not feasible. However, short-term studies have demonstrated a causal relationship between dietary polyphenol intake and improvements in antioxidative and anti-inflammatory activity [120,121,122].

All classes of dietary (poly)phenolics exhibit biological activity. This encompasses all flavonoid subtypes, including flavonols, (iso)flavones, flavanones, anthocyanidins, and flavan-3-ols, as well as dietary non-flavonoid phenolic compounds such as sulforaphane and capsaicin [120]. Accordingly, the following section addresses (poly)phenolics in general.

As with other health-promoting lifestyle practices, polyphenols appear to exert their effects through an adaptive (hormetic) response. For example, direct radical scavenging by polyphenols necessitates concentrations in the millimolar range. However, due to their limited bioavailability, intact polyphenols and their metabolites seldom attain even micromolar levels in systemic circulation. Consequently, polyphenols are likely to induce protective cellular responses rather than directly mediate them [123,124]. Dietary phenolics induce cellular stress by binding to hydrophobic pockets of various proteins, potentially causing denaturation or aggregation [124]. Consequently, upper safe intake limits have been established for certain polyphenols, such as green tea catechins [125]. Additionally, excessive consumption of many spices, including ginger, saffron, and cinnamon, has been associated with toxicity, further supporting their ability to induce cell and tissue stress [126].

The primary response to polyphenol-induced cellular stress is an increase in intracellular ROS production from mitochondria and NADPH oxidases, along with increased NO production from endothelial NOS. Polyphenols are weak radical scavengers, so that their pro-oxidant properties dominate [127,128,129]. The resulting rise in intracellular ROS levels is followed by increases in NAD+ and AMP concentrations, leading to heightened activity of key stress defense mediators such as Nrf2, sirtuins, AMPK, HSF1, and PGC-1α, while simultaneously decreasing NFκB activity. The major outcomes of this response, as previously described, include enhanced antioxidative and anti-inflammatory activity, increased cell repair and regeneration, and improved mitochondrial and vascular function. Additionally, this process strengthens cellular and organism-wide resilience against environmental challenges (Table 1; see refs. [59,121,124,130,131,132,133]).

The ingestion of dietary phenolics along with other healthy lifestyle practices may also exert beneficial effects by targeting the microbiota, micro ribonucleic acid (RNA) pattern, exosome quality, epigenetic modifications in the nucleus, or by modifying brain functions among other regulatory pathways. In any case, a beneficial adaptive response is closely linked to the initial cell and organ stress induced by “healthy” lifestyle practices. In studies where this was analyzed, blocking the initial rise in ROS levels or inhibiting Nrf2 activation in animal models was found to suppress most of the observed health benefits [30,39,59,110,111,128,130,134,135,136]. These findings suggest that ROS and Nrf2 participate in multiple regulatory networks. For example, feeding mice probiotic Lactobacilli led to upregulation of Nrf2 in the liver, improving resistance to oxidative injury [116].

## 8. Acupuncture

Traditional Chinese Medicine has long utilized acupuncture to treat a range of diseases, including chronic inflammatory conditions, acute pain, and ischemic brain damage [137,138,139,140,141]. Numerous clinical trials suggest that acupuncture offers therapeutic benefits. For example, recent meta-analyses of controlled trials in patients with chronic low back pain, herpes zoster infection, stroke-related complications, irritable bowel syndrome, or obesity report positive outcomes associated with acupuncture, but the quality of the trials analyzed is low, often lacking sham-treatment, and firm conclusions therefore are not possible. Indeed, most researchers emphasize the need for high-quality studies to validate these findings (see refs. [26,142,143,144,145,146]). Studies in animals using electroacupuncture have confirmed beneficial effects. There was attenuation of both morphological and functional damage associated with cerebral ischemia, Alzheimer’s disease, and other neurological disorders, as well as hepatic ischemia–reperfusion injury [147,148,149,150,151,152].

Oxidative stress, leading to lipid peroxidation and ferroptosis, is a key contributor to disease progression in these models, while inflammatory mediators exacerbate the damage [147,148,149,150,151,152]. Electroacupuncture has been shown to lower oxidative stress and reduce inflammatory activity. In animal models, the mechanism involves the upregulation of antioxidative enzyme gene expression and enhanced anti-inflammatory mediator levels. Additionally, pharmacological or genetic inhibition of Nrf2 abolishes the beneficial effects of electroacupuncture, suggesting that Nrf2-dependent adaptation plays a central role in its therapeutic impact [147,152,153,154,155]. In the animal models, acupuncture or electroacupuncture also engages sirtuins, AMPK, and PGC-1α activity, contributing to disease mitigation [149,150,152,156,157]. Taken together, findings from animal studies suggest that acupuncture’s healing effects arise from an adaptive response to oxidative stress and inflammation, following the same signaling pathways as exercise (Table 1).

## 9. Cupping Therapy

Cupping therapy has been practiced for centuries in traditional medicine systems across China, India, and the Mediterranean regions as a treatment for pain and inflammation-related conditions. The therapy involves placing several cups on the skin for several minutes, creating a partial vacuum that leads to localized irritation, hyperemia, and tissue stretching. Wet or bleeding cupping involves making small skin incisions to draw blood into the cup [158,159]. However, serious infectious complications have been reported in wet cupping cases [160,161]. Meta-analyses of cupping therapy suggest low to moderate-quality evidence supporting its effectiveness in alleviating neck or back pain, herpes zoster infection, neurodermatitis, and other conditions [159,162,163,164].

While only a few studies have analyzed blood markers, existing data appear to fit with a hormetic concept. For example, significant reductions in IL-6 and tumor necrosis factor-alpha (TNF-α) levels were observed following cupping therapy in healthy athletes before and after exercise [165]. Decreased TNF-α levels were also reported in psoriasis patients undergoing cupping therapy [166]. In an experimental rat model of lipopolysaccharide-induced acute lung injury, cupping therapy reduced tissue damage, improved lung function, and suppressed NFκB activation [167]. This was associated with lower levels of IL-6 and TNF-α, alongside increased levels of anti-inflammatory IL-10. Additionally, these therapeutic effects were accompanied by heightened adenosine levels [167]. Cells secrete the purine nucleoside in response to metabolic or inflammatory stress. Once released, adenosine interacts with adenosine receptors that mediate anti-inflammatory and tissue-protective effects. Interestingly, blocking the adenosine receptor A2B—which is the predominant adenosine receptor in the lungs—abolished the lung-protective effects of cupping therapy. These findings suggest that adenosine plays a central role in mediating the benefits of cupping in this model [167]. There is no direct evidence for a role of Nrf2 in orchestrating the tissue-protective effects of cupping therapy, but activation of adenosine receptors triggers Nrf2-mediated cytoprotection (Table 1) [168,169]. Nonetheless, the involvement of Nrf2 in potential health effects remains hypothetical.

## 10. Discussion

The health claims associated with several of the included practices are supported primarily by clinical trials of low to moderate methodological quality. A potential publication bias favoring studies with positive outcomes cannot be excluded. Nevertheless, all examined practices demonstrate efficacy in relevant animal models, offering insights into the underlying mechanisms of action.

With the exception of exercise and caloric restriction, most practices lack substantial data regarding the potential roles of HIF-1α, PGC-1α, AMPK, and sirtuins in mediating health effects. In contrast, Nrf2 expression has been investigated across nearly all interventions. Although forkhead box transcription factors are presumed to play a role, current literature provides limited insight into their specific involvement. Accordingly, this review concentrates on studies pertaining to Nrf2. As previously noted, inhibition of Nrf2 activation has been attempted in several health practices and was consistently associated with a reduction in health benefits. The selection of traditional non-pharmacological health practices for this comparative analysis was based on the availability of data demonstrating Nrf2 expression, or on evidence strongly suggesting its activation due to the observed oxidative stress and corresponding antioxidant responses. Such data were not identified in the literature concerning yoga, mindfulness-based stress reduction, meditation, breathwork, Tai Chi, Qigong, or massage therapy. Ayurveda was excluded due to the substantial variability in treatment protocols and the diverse composition of herbal extracts employed. As discussed in Section 7, herbs are generally rich in polyphenols and are expected to promote Nrf2 activation. This likely applies to Ayurvedic herbs as well, and indeed, Nrf2 activation has been reported in studies involving Ayurvedic herbal preparations [170,171].

Most health-promoting practices require regular application to elicit sustained benefits. This necessity may be explained by the transient nature of Nrf2 activation, which typically persists for up to 24 h and coincides with a temporary increase in reactive oxygen species, such as after a single bout of exercise. In contrast, repeated exercise induces a more continuous elevation of Nrf2 activity, thereby enhancing long-term cellular defense mechanisms [172,173].

Many health practices may pose risks when applied excessively, such as cardiovascular risks with aerobic exercise, heat or cold treatment, or exposure to hyperbaric or hypobaric conditions [174,175,176,177,178]. Prolonged fasting may cause muscle loss, while consuming high doses of dietary polyphenols may damage the liver [125,126,179]. Cupping therapy may cause severe skin inflammation in the context of infections [160,161]. These observations align well with the concept of hormesis discussed here. Moderate engagement in such practices induces a mild oxidative stress that elicits a beneficial adaptive defense response, whereas excessive activity can overwhelm the organism’s capacity to mount effective protection.

A comparative analysis of health practices beyond their apparent shared induction of oxidative and inflammatory stress with respect to clinical effectiveness was beyond the scope of this review. Such an evaluation may not be feasible due to the considerable heterogeneity in both the practices themselves and the clinical endpoints assessed, which span cardiovascular, neurological, and immunological domains.

## 11. Conclusions

Many health-promoting practices have been observed to cause transient oxidative stress and inflammation, often accompanied by increased sympathetic nervous system activity. These practices include physical exercise, reduced oxygen availability, exposure to heat or cold water, caloric restriction or fasting, consumption of plant phenolics, cupping therapy, and acupuncture. In each of these practices, an adaptive response involving the upregulation of antioxidative and anti-inflammatory activity has been documented. Where studied, activation of Nrf2, sirtuins, and AMPK was consistently observed. In cases involving exercise, dietary polyphenols, acupuncture, and cupping therapy, direct or indirect inhibition of Nrf2 activation was attempted and found to suppress the beneficial effects seen in control groups (Table 2).

Beyond these established pathways, additional molecular mechanisms may contribute to the health benefits of these practices. These could include modifications of the gut microbiome, regulatory circuits in the brain, or epigenetic control of gene expression—areas that require further investigation. It is important to note that the beneficial effects of mild to moderate oxidative or inflammatory stress can be outweighed by detrimental consequences if cellular defense mechanisms are unable to compensate. Toxicity at high doses of stress-inducing agents is a hallmark of the hormesis model [180]. Moreover, excessive or prolonged activation of Nrf2 may disrupt inflammatory and redox signaling pathways, potentially impairing normal organ function [181,182]. Taken together, numerous lifestyle and traditional practices appear to exert beneficial effects through hormetic upregulation of cellular protective and repair mechanisms. This occurs largely through enhanced expression of genes responsible for antioxidative and anti-inflammatory activity, as well as mitochondrial growth. A hormetic health mechanism may not apply to “mild” traditional practices with little physical challenge, such as meditation or breathwork.

## Figures and Tables

**Table 1:** Health practices that elicit largely common adaptive (hormetic) responses. PNS, parasympathetic nervous system; SNS, sympathetic nervous system; TNF-α, tumor necrosis factor-alpha; ?, uncertain; ^a^ Only major signals and mediators are listed.

Lifestyle Practice	Stress Signals ^a^	Adaptive Response Mediators ^a^	Major Improvement
Exposure to heat [62,63,64,65,66,67,68,69,70,71,72]	SNS, ROS, NAD+, AMP	PNS, Nrf2, HSF1, PGC-1α, NFkB	Cardiovascular function
Exposure to cold [72,73,74,75,76,77,78,79,80,81,82,83,84,85,86,87]	SNS, ROS, catecholamines	PNS, Nrf2, sirtuins, AMPK	Cardiovascular function
Hyperbaric oxygen [88,89,90,91,92,93,94,95,96]	ROS	Nrf2, HIF-1α, sirtuins, AMPK	Tissue repair
Exposure to hypoxia [97,98,99,100,101,102]	SNS, ROS, NO, NAD^+^, AMP	HIF-1α, Nrf2, PGC-1α, AMPK, sirtuins	Vascular + cognitive function
Caloric restriction [103,104,105,106,107,108,109,110,111,112,113,114,115,116,117,118,119]	ROS, NAD+, AMP	Nrf2, AMPK, PGC-1α, sirtuins	Cardiometabolic function
Dietary polyphenols [120,121,122,123,124,125,126,127,128,129,130,131,132,133,134,135,136]	ROS, NO, NAD+, AMP	Nrf2, AMPK, PGC-1α, sirtuins	Organ function
(Electro) acupuncture [137,138,139,140,141,142,143,144,145,146,147,148,149,150,151,152,153,154,155,156,157]	ROS	Nrf2, sirtuins, AMPK	Tissue function
Cupping therapy [158,159,160,161,162,163,164,165,166,167,168,169]	IL-6, TNF-α, adenosine	Nrf2 (?)	Anti-inflammatory response

**Table 2:** Key messages.

Key Messages
Similar to exercise, various other health-promoting practices induce transient oxidative and inflammatory stress, leading to moderate damage to cellular and tissue constituents. These practices include hot or cold treatment, exposure to hyper- or hypobaric oxygen, caloric restriction, dietary polyphenols, cupping therapy, and acupuncture.
Following the initial damage, an adaptive (hormetic) response is induced, characterized by the upregulation of antioxidative, anti-inflammatory, and cell repair activities, as well as enhancing mitochondrial function.
This adaptive response to initial damage drives the health benefits, which may extend throughout the body, promoting an improved functional state and greater resilience against future challenges.
A key pathway involves the initial rise in oxygen radical and nitric formation, which triggers translocation of the redox-sensitive transcription factor Nrf2 to the nucleus, leading to the increased expression of hundreds of genes involved in cell defense, repair, and regeneration.
Additional signaling pathways may further enhance health outcomes, which include NAD+/sirtuins, AMP/AMPK, and PGC-1α.
Attempts to suppress initial damage or inhibit Nrf2 activation have been made in some health practices and have been shown to diminish their beneficial effects.
Actively challenging the body’s physiological balance appears to be an effective strategy for improving overall function and increasing resistance to future stressors.

## Data Availability

No new data were created or analyzed in this study. Data sharing is not applicable to this article.

## Abbreviations

The following abbreviations are used in this manuscript:

ADP	adenosine diphosphate
AMP	adenosine monophosphate
AMPK	adenosine monophosphate-activated protein kinase
Anti-inflamm.	anti-inflammatory
Anti-ox	antioxidative
ARE	antioxidant response element
ATP	adenosine triphosphate
Cul3	Cullin 3-based E3 ubiquitin ligase
detox	detoxifying
DNA	deoxyribonucleic acid
ecNOX4	endothelial cell nicotinamide adenine dinucleotide phosphate oxidase 4
ETC	electron transfer chain
FFA	free fatty acids
H_2_O_2_	hydrogen peroxide
HIF-1α	hypoxia-inducible factor 1 alpha
HSF1	heat shock factor 1
Hsp	heat shock protein
IL	interleukin
IL-1RA	interleukin-1 receptor antagonist
Inflam.	inflammatory
Keap1	Kelch-like ECH-associated protein-1
Mitochond.	mitochondrial
NAD+	nicotinamide adenine dinucleotide
NADPH	nicotinamide adenine dinucleotide phosphate
NFκB	nuclear factor kappa light chain-enhancer of activated B cells
nNOS	neuronal nitric oxide synthase
NO	nitric oxide
NOS	nitric oxide synthase
NOX	nicotinamide adenine dinucleotide phosphate oxidase
Nrf2	nuclear factor erythroid 2-related factor 2
NS	nervous system
PGC-1α	peroxisome proliferator-activated receptor gamma coactivator-1 alpha
PNS	parasympathetic nervous system
p38	protein 38
p62	protein 62
RNA	ribonucleic acid
ROS	reactive oxygen species
SH	sulfhydryl
sirtuin	silencing information regulator 2-related enzyme
sMAF	small musculoaponeurotic fibrosarcoma protein
SNS	sympathetic nervous system
TNF-α	tumor necrosis factor-alpha
XO	xanthine oxidase
Vit	vitamin
